# Characterization of Elusive Reaction Intermediates Using Infrared Ion Spectroscopy: Application to the Experimental Characterization of Glycosyl Cations

**DOI:** 10.1021/acs.accounts.2c00040

**Published:** 2022-05-26

**Authors:** Floor ter Braak, Hidde Elferink, Kas J. Houthuijs, Jos Oomens, Jonathan Martens, Thomas J. Boltje

**Affiliations:** † Radboud University, Institute for Molecules and Materials, Synthetic Organic Chemistry, Heyendaalseweg 135, 6525 AJ Nijmegen, The Netherlands; ‡ Radboud University, FELIX Laboratory, Institute of Molecules and Materials, Toernooiveld 7, 6525 ED Nijmegen, The Netherlands

## Abstract

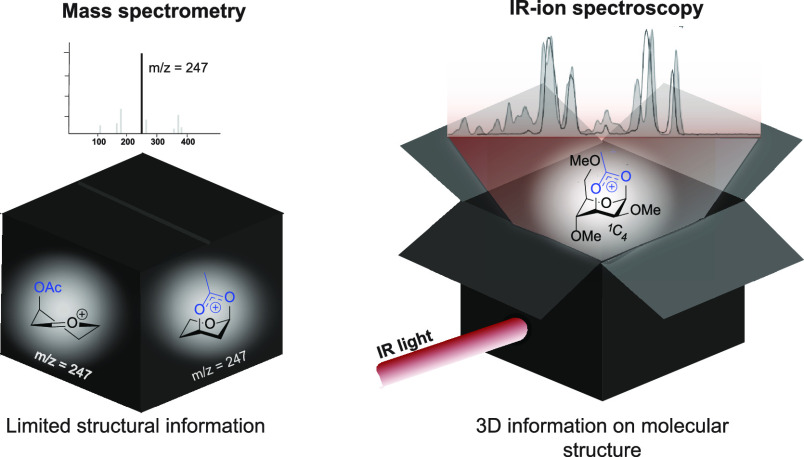

A detailed understanding of the reaction mechanism(s) leading to stereoselective product formation is crucial to understanding and predicting product formation and driving the development of new synthetic methodology. One way to improve our understanding of reaction mechanisms is to characterize the reaction intermediates involved in product formation. Because these intermediates are reactive, they are often unstable and therefore difficult to characterize using experimental techniques. For example, glycosylation reactions are critical steps in the chemical synthesis of oligosaccharides and need to be stereoselective to provide the desired α- or β-diastereomer. It remains challenging to predict and control the stereochemical outcome of glycosylation reactions, and their reaction mechanisms remain a hotly debated topic. In most cases, glycosylation reactions take place via reaction mechanisms in the continuum between S_N_1- and S_N_2-like pathways. S_N_2-like pathways proceeding via the displacement of a contact ion pair are relatively well understood because the reaction intermediates involved can be characterized by low-temperature NMR spectroscopy. In contrast, the S_N_1-like pathways proceeding via the solvent-separated ion pair, also known as the glycosyl cation, are poorly understood. S_N_1-like pathways are more challenging to investigate because the glycosyl cation intermediates involved are highly reactive. The highly reactive nature of glycosyl cations complicates their characterization because they have a short lifetime and rapidly equilibrate with the corresponding contact ion pair. To overcome this hurdle and enable the study of glycosyl cation stability and structure, they can be generated in a mass spectrometer in the absence of a solvent and counterion in the gas phase. The ease of formation, stability, and fragmentation of glycosyl cations have been studied using mass spectrometry (MS). However, MS alone provides little information about the structure of glycosyl cations. By combining mass spectrometry (MS) with infrared ion spectroscopy (IRIS), the determination of the gas-phase structures of glycosyl cations has been achieved. IRIS enables the recording of gas-phase infrared spectra of glycosyl cations, which can be assigned by matching to reference spectra predicted from quantum chemically calculated vibrational spectra. Here, we review the experimental setups that enable IRIS of glycosyl cations and discuss the various glycosyl cations that have been characterized to date. The structure of glycosyl cations depends on the relative configuration and structure of the monosaccharide substituents, which can influence the structure through both steric and electronic effects. The scope and relevance of gas-phase glycosyl cation structures in relation to their corresponding condensed-phase structures are also discussed. We expect that the workflow reviewed here to study glycosyl cation structure and reactivity can be extended to many other reaction types involving difficult-to-characterize ionic intermediates.

## Key References

- ElferinkH.; SeverijnenM. E.; MartensJ.; MensinkR. A.; BerdenG.; OomensJ.; RutjesF. P. J. T; RijsA. M.; BoltjeT. J.Direct Experimental Characterization of Glycosyl Cations by Infrared Ion Spectroscopy. J. Am. Chem. Soc.2018, 140, 6034–603810.1021/jacs.8b0123629656643PMC5958338.^1^ The first report on the characterization of glycosyl cations using infrared ion spectroscopy.
- ElferinkH.; MensinkR. A.; CastelijnsW. W.; JansenO.; BruekersJ. P.; MartensJ.; OomensJ.; RijsA. M.; BoltjeT. J.The Glycosylation Mechanisms of 6,3-Uronic Acid Lactones. Angew. Chem., Int. Ed.2019, 58, 8746–875110.1002/anie.20190250731017713.^2^ A mechanistic study of the reaction mechanism(s) of glycosyl cations derived from conformationally locked uronic acids.
- HansenT.; ElferinkH.; van HengstJ. M. A.; HouthuijsK. J.; RemmerswaalW. A.; KrommA.; BerdenG.; van der VormS.; RijsA. M.; OverkleeftH. S.; FilippovD. V.; RutjesF. P. J. T.; van der MarelG. A.; MartensJ.; OomensJ.; CodeeJ. D. C.; BoltjeT. J.Characterization of glycosyl dioxolenium ions and their role in glycosylation reactions. Nat. Commun.2020, 11, 266410.1038/s41467-020-16362-x32471982PMC7260182.^3^ A systematic study of the ability of acyl groups to participate in the stabilization of glycosyl cations.

## Introduction

Glycosylation, the expression of carbohydrate structures (glycans) on proteins and lipids, is found in all domains of life.^4^ The collection of all glycans found on a cell is called the “glycome”, which is rich in information and a key player in a plethora of physiological and pathological processes.^5^ The information contained within the glycome can be written, read, and erased by glycosyltransferases, lectins, and glycosidases, respectively. Glycans are structurally very diverse because they are composed of different monosaccharides which, when connected, give rise to different regio- and stereoisomers producing long, short, branched, and linear glycans that can attach to a protein and/or lipid carrier.^6^ Similar to genomics for DNA and proteomics for proteins, “glycomics” is the study that seeks to identify and understand the structure and function of specific glycans in biological processes. Genomics and proteomics have benefited from the availability of advanced molecular biology methods and the availability of well-defined synthetic standards prepared using automated solid-phase synthesis methodology. Because their biosynthesis is not under direct genetic control, glycans are expressed in microheterogeneous forms, challenging their isolation and characterization from a biological sample. Hence, in many cases well-defined oligosaccharides can be obtained only by chemical or enzymatic synthesis.^7^ The chemical synthesis of glycans is challenging because of the structural diversity and complexity of this class of molecules. The monosaccharide constituents of glycans are connected to each other at the anomeric center via an acetal linkage termed the glycosidic bond. Glycosidic bonds can exist as two anomers (equatorial and axial), and the anomeric stereochemistry is usually defined relative to the C-2 substituent, 1,2-*cis* or 1,2-*trans*, or relative to the last chiral substituent on the carbohydrate chain (α or β).^8^

**Scheme 1:**
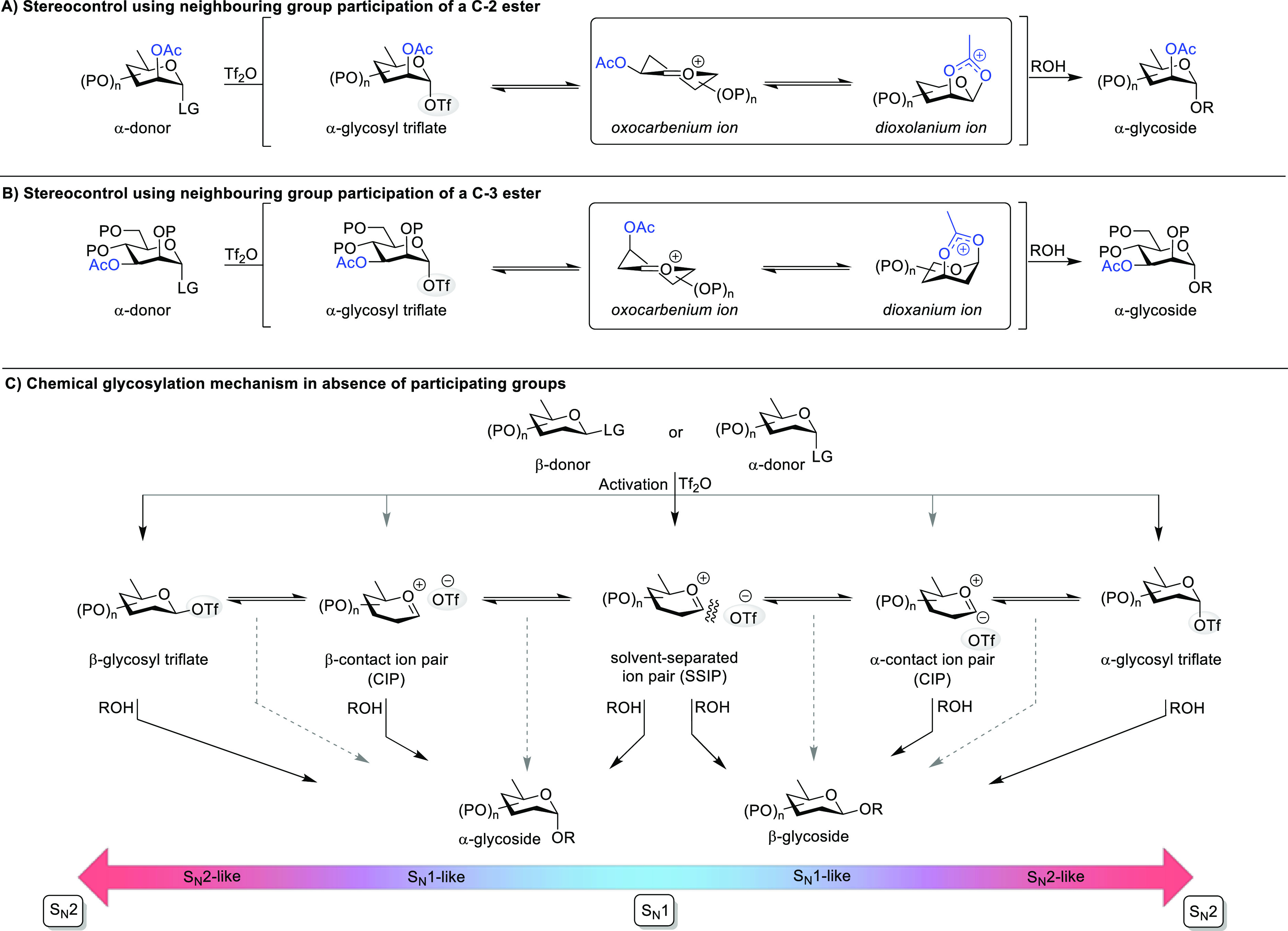
Stereoselective Glycosylation via (A) the Neighboring Group Participation of a C-2 Acyl Group and (B) the Neighboring Group Participation of a C-3 Acyl Group and (C) the Glycosylation Mechanisms of Glycosyl Triflate Intermediates

Glycosidic bonds are synthesized in so-called glycosylation reactions, which can be described as a nucleophilic substitution reaction between a glycosyl donor carrying an anomeric leaving group and a glycosyl acceptor containing a nucleophilic alcohol. Controlling the diastereoselectivity of glycosylation reactions is the major challenge in oligosaccharide synthesis and is achieved by the application of two main strategies.^9^ First, an acyl group at the C-2 position of the glycosyl donor can be used to trap the glycosyl cation formed after the departure of the leaving group, affording a dioxolanium ion (Scheme 1A). The displacement of this dioxolanium ion by the glycosyl acceptor affords 1,2-*trans* glycosides with high stereoselectivity.^10^ In general, the use of a 2-*O*-acyl functionality to synthesize 1,2-*trans* glycosides is very reliable and highly stereoselective and can be applied to solid-phase oligosaccharide synthesis.^11^ In the case of d-gluco-type donors, β-linked products are obtained, whereas d-manno-type donors give α-linked products. Furthermore, this strategy is also applicable to the synthesis of 2-deoxy-2-amino-glycosides using amine-protecting groups that can engage in neighboring group participation (NGP). The participation of acyl functionalities further away from the anomeric center, i.e., placed on the C-3, C-4, or C-6 hydroxyl groups, has also been suggested to direct the stereoselectivity of glycosylation reactions (Scheme 1B). This potentially allows for the utilization of the relative stereochemistry of C-3, C-4, or C-6 groups to control the facial selectivity in glycosylation reactions, thereby enabling the stereoselective synthesis of C-2-deoxy and 1,2-*cis*-glycosides. However, contradictory results have been reported, and there is an ongoing debate as to the role and strength of this stereoelectronic effect.^12^

The second main strategy is utilizing glycosyl donors that do not contain protecting groups capable of participation (Scheme 1C).^13,14^ In this case, the glycosyl cation is trapped by the triflate counterion, resulting from most promoter systems, leading to an α- or β-glycosyl triflate. The mechanism of glycosylation reactions proceeding via these intermediates continues to be a topic of much research and takes place in the continuum between S_N_1-like and S_N_2-like reaction pathways.^15^ Reactions with the glycosyl acceptor can take place via a dissociative (S_N_1) or associative (S_N_2) substitution reaction. Glycosyl triflates can be characterized by low-temperature NMR and have been shown to be reactive intermediates via S_N_2-like pathways, leading to stereospecific reactions affording the opposite diastereomer as the main product. In some cases, the observed glycosyl triflate affords a product with retention of the anomeric configuration, which cannot be explained by an S_N_2-like pathway. In these cases, reactions are expected to take place via other reaction intermediates that are in rapid equilibrium with the observed α-glycosyl triflate following the Curtin–Hammett principle. Likely candidates in this respect are the glycosyl oxocarbenium ion and β-glycosyl triflate (Scheme 1C).

Hence, to better understand the mechanistic pathways of glycosylation reactions, identification of the structures of all reactive intermediates is crucial. However, the intrinsic high reactivity, short lifetime, and equilibrium with the corresponding contact ion pair complicates the characterization of low-abundance reactive intermediates. This challenge has recently been addressed by trapping the oxocarbenium ion in a superacidic medium, which enabled the recording of NMR spectra of glycosyl cations derived from 3,4,6-tri-*O*-acetyl-2-deoxy-glucosides.^16^ Under superacidic conditions, all acetyl groups were protonated, thereby disabling the assessment of their ability to stabilize the cationic center. Glycosyl cations have also been generated using mass spectrometry in the absence of solvent and counterions, offering unique conditions for studying the stability and reactivity of these intermediates.^17−20^ Although the information that can be extracted from a single mass measurement is limited, various forms of tandem mass spectrometry provide a means of determining the structural details. Infrared ion spectroscopy (IRIS) has emerged as a powerful method for characterizing molecular ions in the gas phase, and we and others have applied this technique to elucidate the structure of glycosyl cations.^1,21^

Herein, we review the recent developments in the use of mass spectrometry to generate glycosyl cations and the use of IRIS to characterize them spectroscopically. The main types of instrumentation will be discussed, including their capabilities, differences, and limitations. An overview of glycosyl cations characterized thus far using IRIS is provided. The structural insights from IRIS applied to glycosyl ion structure is discussed along with its relevance to glycosyl cations as reactive intermediates in the condensed phase. We note that in parallel there have been strong efforts to apply IRIS in the chemical analysis of glycans, but this is beyond the scope of the present review.^22−27^

### Investigating Glycosyl Cations Using Mass Spectrometry

**Figure 1:**
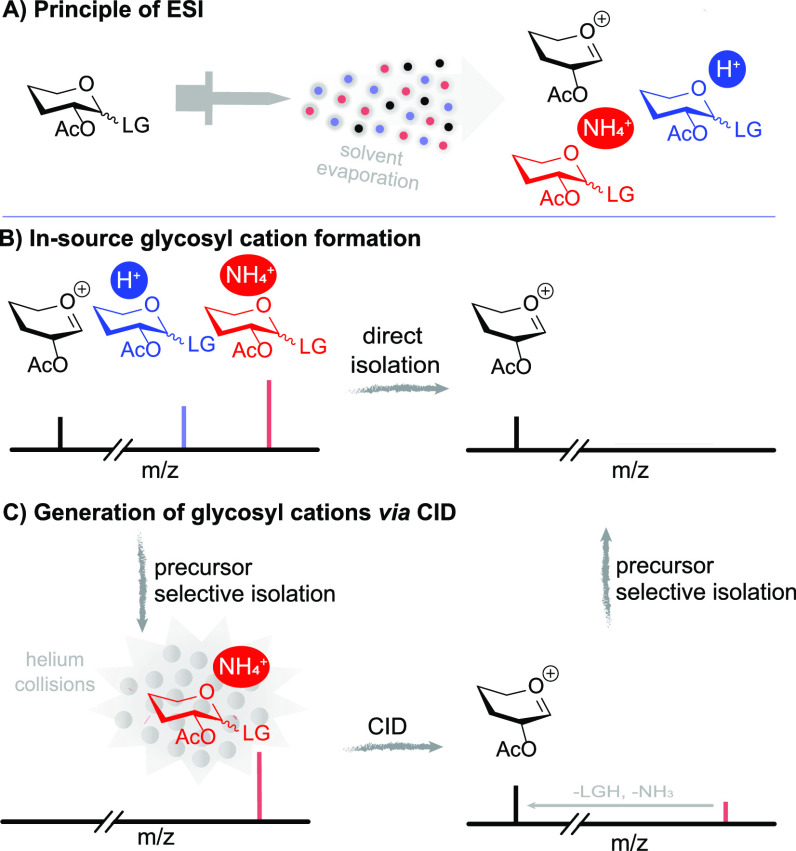
(A) Principle of electrospray ionization. (B) Direct in-source formation of a glycosyl cation. (C) Formation of a glycosyl cation through CID.

The introduction of soft-ionization sources for mass spectrometry, in particular, electrospray ionization (ESI) and matrix-assisted laser desorption ionization (MALDI), enabled gas-phase studies of intact labile molecular ions using mass spectrometry.^28,29^ The ESI process is often referred to as a means to directly transfer ions from solution to the gas phase and is now used extensively to study systems ranging from small molecules to entire protein complexes (Figure 1A).^30,31^ Such gas-phase studies offer a unique environment for examining factors influencing the structure, stability, and reactivity of glycosyl cations under controlled, isolated conditions. Although the amount of structural information that can be extracted from a mass-to-charge ratio (*m*/*z*) measurement is limited, various forms of tandem mass spectrometry (MS/MS) have been used to complement *m*/*z* information with indirect structural and reactivity information. Tandem mass spectrometry involves the activation of a mass-selected ion population in order to induce fragmentation reactions, which are often unique for different molecular species that are inseparable on the basis of *m*/*z*.^32,33^ Here we will focus on “in-source” fragmentation, where all ions are accelerated and collisionally activated in a region of the mass spectrometer where high-vacuum conditions are not yet reached (Figure 1B), and mass-isolated collision-induced dissociation (CID), where ions of a specific mass are selected and fragmented separately from ions of other masses (Figure 1C). CID has the advantage that mass isolation prior to fragmentation allows for the direct correlation of fragment ions to an individual precursor ion.

**Scheme 2:**
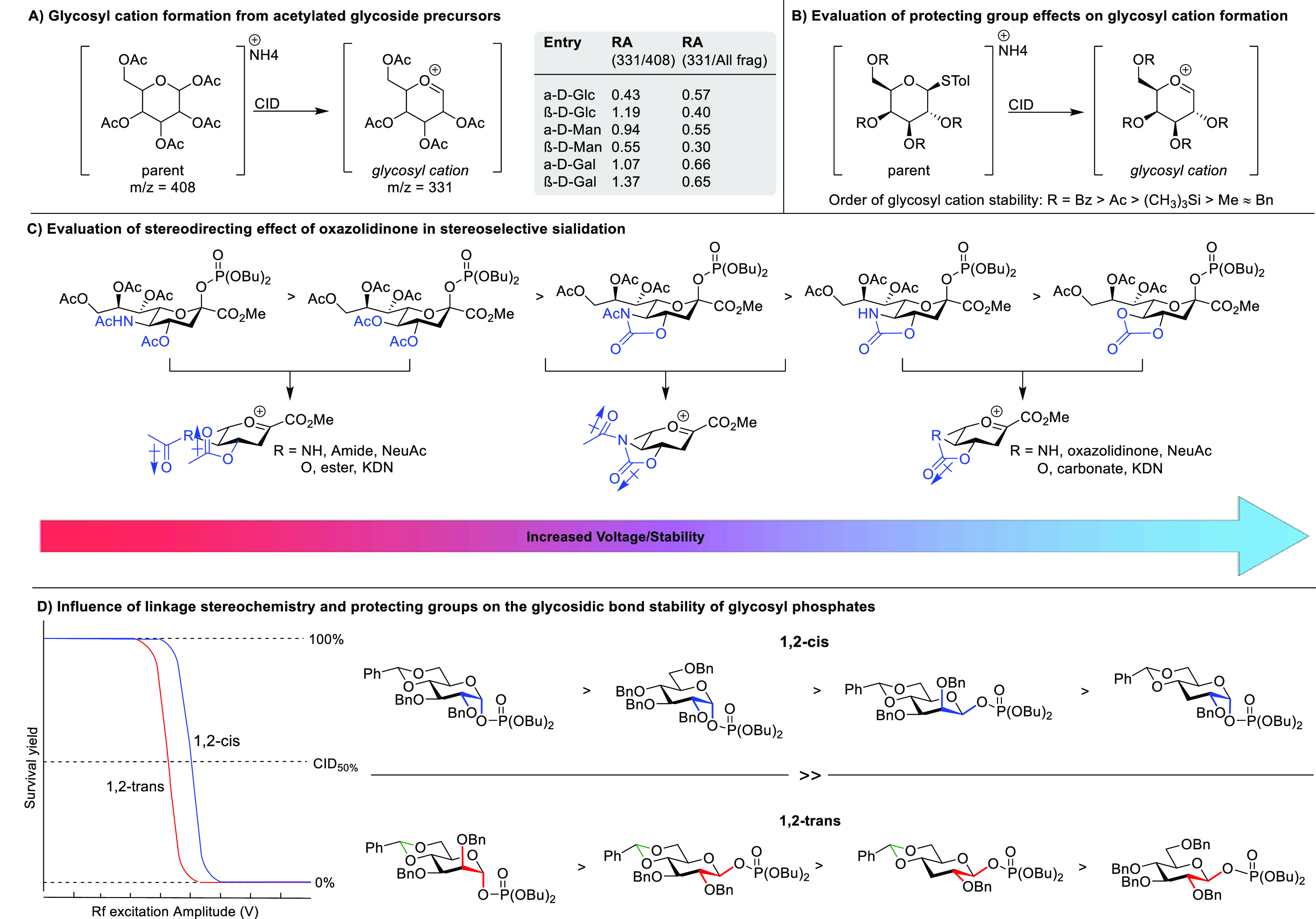
(A) Formation of glycosyl cations from acetylated glycoside precursors using CID (RA = Relative Abundance of the Glycosyl Cation Fragment), (B) Influence of the Protecting Group Nature on Glycosyl Cation Formation, (C) Influence of Protecting Groups and Their Dipole Moment on the Energetics of Sialyl Oxocarbenium Ions, and (D) Evaluation of the Effects of Linkage Stereochemistry, Leaving Group Geometry, and Protecting Group Pattern on the Stability of the Glycosyl Linkage Using Survival Yield Analysis

In 2005, Denekamp and co-workers performed pioneering mass spectrometry experiments investigating the generation and stability of glycosyl cations. Peracetylated α- and β-hexoses were ionized and fragmented using CID.^17^ Peracetylated β-anomers with a 1,2-*trans* relative stereochemistry most readily underwent fragmentation toward a glycosyl cation, presumably resulting from the departure of the anomeric leaving group. This high reactivity was attributed to the ability of the C-2 ester to directly perform neighboring group participation in this specific configuration (Scheme 2A). Additionally, the relative stereochemistry at C-4 was investigated, with galactose fragmenting more readily to the glycosyl ion than glucose and mannose. In subsequent work, the same authors evaluated the effect of the nature of the protecting and leaving groups on glycosyl cation formation and the stabilization of a series of glycopyranosyl thioglycosides (Scheme 2B).^18^ Glycosyl cations were observed after CID of the parent ammonium adducts bearing protecting groups capable of NGP (Bz or Ac). Despite their electron-withdrawing character, these acyl groups were shown to stabilize the oxocarbenium ion effectively through π-overlap. Conversely, the presence of ether protecting groups lacking this favorable overlap was shown to hamper glycosyl cation formation. The ease of glycosyl cation formation correlated with the protecting group pattern used at the C-2 and/or C-4 position and was found to be Bz > Ac > (CH_3_)_3_Si > alkyl. When a series of thioaryl leaving groups were compared, only a modest effect on glycosyl cation formation was found.

Crich et al. used the in-source fragmentation of sialoside donors to produce the corresponding glycosyl cations to measure sialyl donor reactivity (Scheme 2C).^19^ To this end, threshold fragmentation energies of a series of sialoside donors carrying 4,5-*N*-acetyl oxazolidinone, 4,5-oxazolidinone, or carbonate protecting groups were investigated. The use of cyclic protecting groups necessitated higher excitation energies (in-source cone voltage) to induce fragmentation toward the glycosyl cation. This effect was attributed to the electron-withdrawing abilities of the oxazolidinone and cyclic carbonates by the alignment of a single large dipole antiparallel to the mean plane of the pyranose ring. Hence, it was concluded that cyclic protecting groups retarded the formation of the glycosyl cation and instead promoted associative reaction mechanisms.

More recently, Rodgers et al. studied the influence of the anomeric configuration and protecting group pattern on the stability of the glycosidic bond.^20^ To this end, the sodium adducts of eight glycosyl phosphates were examined via survival yield analysis based on their CID fragmentation toward glycosyl cations (Scheme 2D). They found that the relative C1–C2 stereochemistry is a major factor affecting the stability of the glycosidic bond. Greater stability was found for 1,2-cis anomers than for their respective 1,2-trans anomers. The glycosidic bond cleavage of 1,2-*cis*-glycosyl phosphates was therefore hypothesized to proceed via an oxocarbenium ion intermediate, whereas the cleavage of 1,2*-*trans isomers takes place via a syn elimination mechanism akin to a McLafferty type rearrangement. Furthermore, it was found that cyclic protecting groups stabilize the glycosidic bond of 1,2-cis anomers while activating the bond of the 1,2-trans anomers. The same effect was found when a C-3 BnO substituent is present, whereas no significant effect on bond stability was found for the C-2 BnO substituent.

Although such gas-phase MS studies provide interesting fundamental observations that may suggest aspects of reactivity that can be extended to the condensed phase, a major limitation is that no direct structural information is obtained for the glycosyl cations produced. Generating a fundamental understanding of the underlying chemical reactivity requires a clearer picture of the glycosyl cation structure. The use of MS in combination with IRIS has therefore emerged as a powerful method for assigning molecular structures to ions observed in MS experiments.^26,27,34−38^ In this Account, we focus on the extra dimension that is obtained by combining MS with IR spectroscopy in search of a better understanding of the chemical glycosylation mechanism.

### Infrared Ion Spectroscopy (IRIS)

The challenge to obtaining IR spectra of gaseous, mass-selected molecular ions lies in the extremely low densities of ions in any type of tandem mass spectrometer (≪10^6^ cm^–3^), which precludes the application of direct absorption spectroscopy using conventional (FT)IR spectrometers. Various action spectroscopy methods have been developed to overcome these challenges.

**Figure 2:**
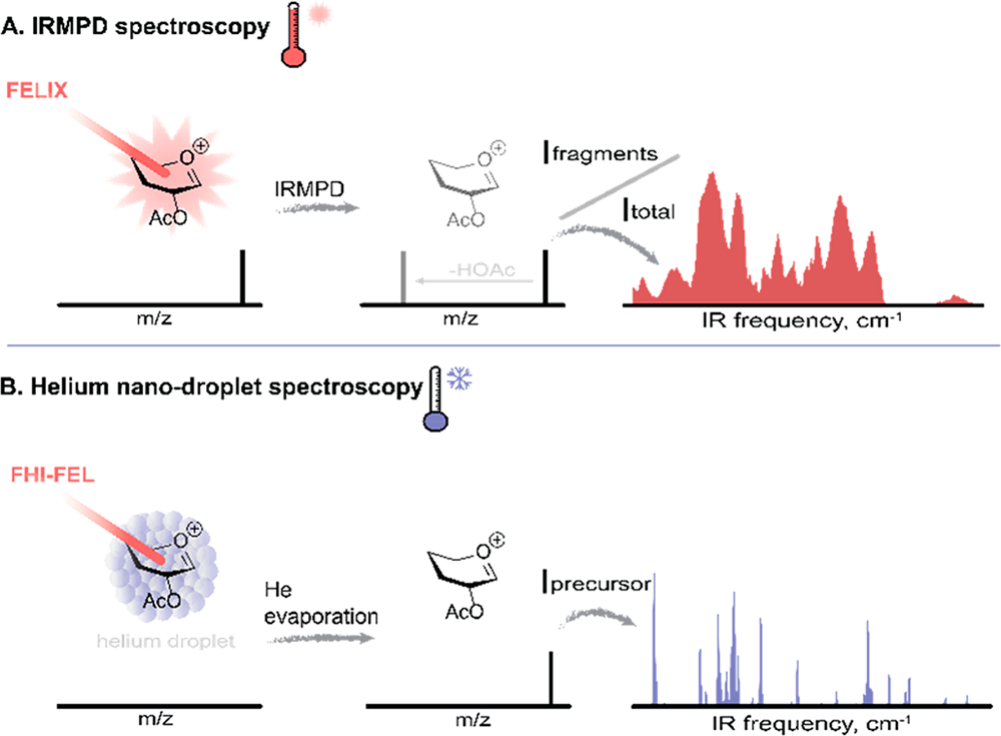
(A) Principle of IRMPD spectroscopy at ambient temperature. (B) Principle of helium nanodroplet spectroscopy at 0.37 K.

IR multiple-photon dissociation (IRMPD) spectroscopy was originally developed in the early 1990s by employing Fourier transform ion cyclotron resonance (FTICR) mass spectrometers and CO_2_ lasers that were line-tunable at wavelengths between 9 and 11 μm (Figure 2A).^39−41^ Irradiating the mass-selected ion cloud inside the ICR cell while the laser frequency is being tuned induces precursor ion dissociation whenever the laser frequency is resonant with a vibrational band of the investigated ion. Simultaneously, a series of mass spectra are recorded to enable the detection of ion fragmentation. By plotting the fractional ion dissociation as a function of laser frequency, an IR spectrum can be reconstructed (Figure 2A). Because the dissociation threshold is much higher than the photon energy, sizable laser powers are required to drive multiple-photon absorption. Because of the limited analytical usefulness of the CO_2_ laser wavelength range, the technique would likely have fallen into oblivion if it were not for the advent of widely tunable IR lasers in the early 2000s, in particular, IR free-electron lasers (FELs) and OPO/OPA systems. Currently, IR FEL facilities at Radboud University (FELIX), Université Paris-Sud (CLIO), and the Fritz-Haber Institute (FHI-FEL) are routinely used for ion spectroscopy in the fingerprint IR range (∼500 to 2000 cm^–1^), and many groups employ table-top OPO sources to cover the X–H stretching range between 2500 and 4000 cm^–1^. The IRMPD process relies on rapid intramolecular vibrational redistribution (IVR) during the IR-induced activation of the ion. The gradual heating of the system during the sequential absorption of multiple photons typically causes a small red shift (a few cm^–1^) and broadening of the vibrational band as compared to a linear absorption spectrum.^42,43^

To mitigate the band broadening associated with IRMPD spectroscopy, linear (one-photon) action spectroscopy can be achieved by attaching a weakly bound tag to the species under investigation that serves as a “messenger” reporting on the absorption of an IR photon.^44^ The binding energy of the tag should be lower than the IR photon energy, and the tag should minimally alter the IR fingerprint of the analyte, making rare gas (Rg) atoms favorable tags. To stabilize the [M + Rg]^+^ ion, cryogenic temperatures are required. Resonant excitation of the [M + Rg]^+^ ion by a single IR photon induces tag detachment, which is monitored in the MS as an *m*/*z* shift. Even lower temperature spectroscopy can be achieved using He nanodroplets, which are clusters of thousands of He atoms that have been employed for spectroscopic experiments since the early 1990s (Figure 2B).^45−47^ The He nanodroplets are at 0.37 K, and their superfluidity provides the ultimate noninteracting matrix environment. The droplets are produced in a supersonic expansion through a cold nozzle and can contain between 10^2^ and 10^6^ He atoms depending on the backing pressure and nozzle temperature. The droplets pick up gaseous molecules that they encounter, and these analytes quickly thermalize to 0.37 K by the evaporation of He atoms, thus becoming embedded within the He droplet. Various methods have been devised to study the spectroscopy of ionized molecules embedded in He droplets.^48,49^ In the implementation of von Helden and co-workers, a pulsed nozzle at 15–25 K produces He droplets in the size range of 10^4^–10^6^ atoms.^25^ They travel through a linear hexapole RF ion trap at 80 K containing the thermalized and mass-selected ions of interest. In the trap, droplets pick up an ion and continue their journey toward the extraction zone of a time-of-flight mass spectrometer (TOF-MS). Here, an FEL pulse irradiates the droplets, and resonant absorption by the embedded ion causes its ejection from the droplet and its detection in the TOF-MS. The measured spectra sample the ion at very low temperature, greatly reducing the number of quantum states and conformations populated, and typically provide better spectral resolution than IRMPD spectra (Figure 2B).

To extract information on molecular structure from experimental IR spectra, they can be matched to reference spectra either measured from chemical standards or predicted from quantum chemically calculated vibrational spectra. Workflows to generate predicted IR spectra typically involve a large set of candidate geometries for a specific glycosyl cation isomer that reflects all possible conformations and modes of intramolecular stabilization.^50,51^ After a low-level geometry optimization, often using molecular mechanics, a number of low-energy structures are selected. The selected geometries are then optimized at the density functional theory (DFT) level, and their predicted IR spectra and Gibbs free energies are computed. Electronic energies are usually also computed at higher levels of theory to give more accurate relative energies. Predicted IR spectra of the lowest-energy conformations are then compared to the experimental spectrum, facilitating the structural assignment of the glycosyl cation. Especially for comparison with IRMPD spectra, assignments are mainly based on peak positions (cm^–1^) because IRMPD band intensities may deviate somewhat from computed linear IR intensities.

### Characterization of Glycosyl Cations Using IRIS

**Figure 3:**
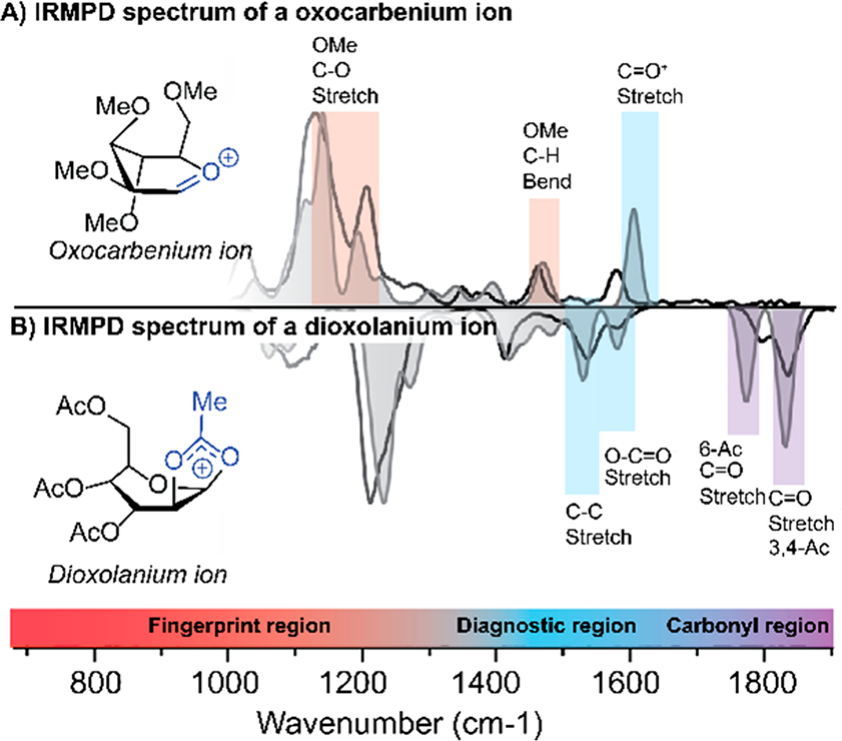
Comparison of computed IR spectra (filled gray) and measured IRMPD spectra (black line). IRMPD spectra of (A) a mannosyl oxocarbenium ion and (B) a mannosyl dioxolanium ion.

Our first IRIS-based characterization of glycosyl cations potentially involved in glycosylation reactions focused on glycosyl cations that were generated by CID MS/MS from mannosides modified with methyl (Figure 3A) or acetyl (Figure 3B) protecting groups.^1^ In the case of the permethylated mannoside, the IR spectrum showed a vibrational characteristic for the anomeric carbonylonium C=O^+^–C stretch (∼1609 cm^–1^, Figure 3A). The experimental spectrum (Figure 3A, black line) could be matched to the DFT-calculated spectrum (Figure 3A, color fill) of the mannosyl oxocarbenium ion in the ^3^E conformation.

In contrast, a mannoside donor modified with acetyl protecting groups formed a glycosyl dioxolanium ion by the participation of the C-2 acetyl group (Figure 3B). This was observed from the IR spectrum as the interaction of a participating group with the anomeric center leading to the disappearance of the anomeric carbonylonium C=O^+^ stretch. Instead, O–C^+^–O and C^+^–CH_3_ stretching modes (∼1540 and ∼1495 cm^–1^) characteristic of the formation of a bicyclic glycosyl dioxolanium ion were observed. C=O stretching vibrations of the nonparticipating acetyl esters appeared at higher wavenumbers (1700–1800 cm^–1^). At lower wavenumbers (700–1500 cm^–1^), a large number of bands were observed, including many that are diagnostic in deciding on the best match of the experimental (Figure 3B, black line) versus DFT calculated spectrum (Figure 3B, color fill).

The first example of helium nanodroplet spectroscopy to characterize glycosyl cations was reported by Pagel et al. and presents the IRIS spectra of glycosyl cations originating from gluco-, galacto-, and mannosides containing a C-2 acetyl group.^21^ NGP of the C-2 ester was confirmed for all three glycosyl cations as well as finer structural details observable as a result of the exquisite spectral resolution of the helium nanodroplet method.^21^ However, the structural assignment of the galactosyl cation was impeded by a more congested fingerprint region presumable due to coexisting dioxolanium ion conformers. Coexisting ring conformations in the unprotected galactosyl cations were further explored by Dvores et al. by combining IRMPD spectroscopy with more advanced computational approaches.^52^ Their simulations were unable to definitively assign the oxocarbenium ion but did indicate that a rapid conversion between ring conformations should occur at room temperature.

**Scheme 3:**
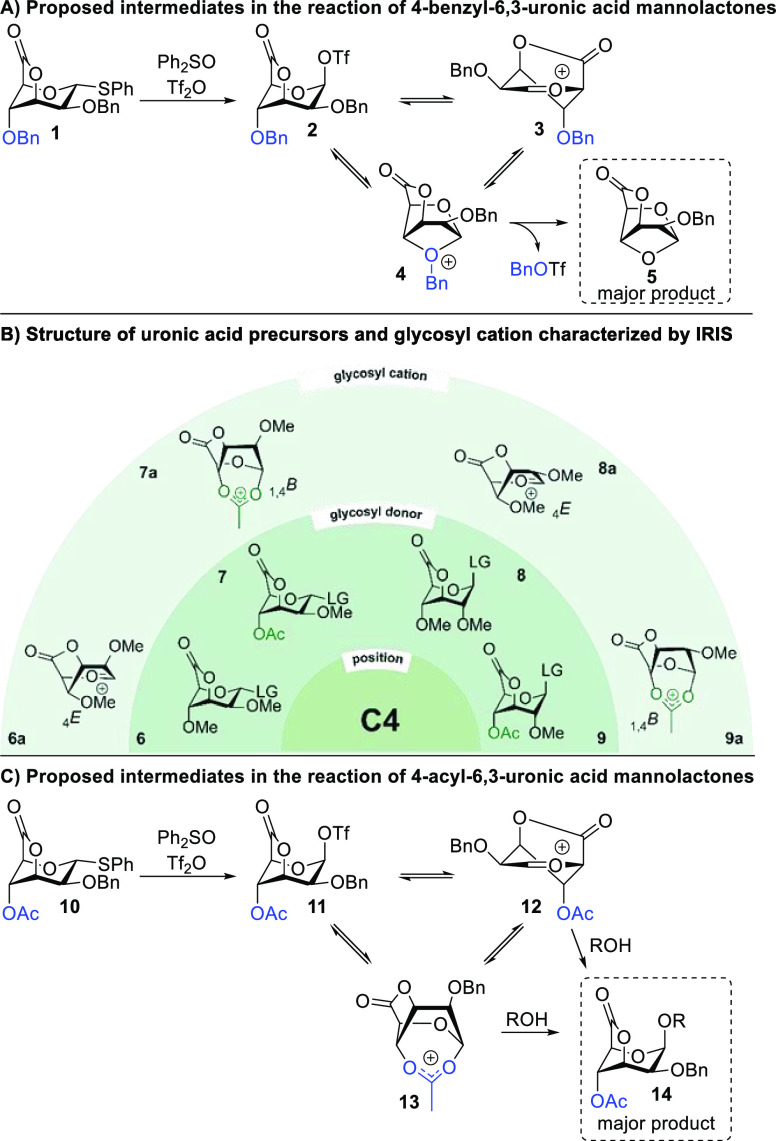
(A) Proposed Intermediates in the Reaction of 4-Benzyl-6,3-uronic Acid Mannolactones, (B) Overview of Characterized Glycosyl Cations Derived from 6,3-Uronic Acid Lactone Donors (**6** and **8**, LG = SOPh; **7** and **9**, LG = SPh), and (C) Proposed Intermediates in the Reaction of 4-Acetyl-6,3-uronic Acid Mannolactones

Subsequent studies focused on probing the contribution of protecting groups at more remote positions in shaping glycosyl cation structures. We explored the use of IRIS to investigate glycosylation reactions of 6,3-uronic acid lactones. We previously observed that conformationally locked 6,3-mannuronic acid lactones reacted with very high β-selectivity.^2,53^ However, glycosyl donors carrying a C-4 benzyl substituent (**1**) provided β-glycosides in very low yields because a 1,4-anhydrosugar (**5**) was formed as the major product (Scheme 3A).^53^ The lactone bridge presumably leads to β-selective oxocarbenium ion conformer **3** but also allows for the participation of the C-4 benzyl ether (**4**), which upon loss of benzyl triflate affords 1,4-anhydrosugar **5** (Scheme 3A). To prevent anhydrosugar formation, we prepared donors **6**–**9** carrying a C-4 *O*-acetyl or *O*-methyl group (Scheme 3B).^2^ The structures of the corresponding glycosyl cations were determined using IRIS. The thioglycoside leaving group was oxidized to the sulfoxide in the case of **6** and **8** to avoid overlap in fragmentation channels. Cations resulting from methylated uronic acid lactones **7** and **9** were characterized as oxocarbenium ions, even though the calculated minimum-energy conformation involved the participation of the C-4 *O*-methyl group (Scheme 3B). In the case of the C-4 *O*-acetyl-modified donors (**7** and **9**), stabilization of the cationic center by the C-4 acetyl group was observed (Scheme 3B).^2^ The absence of solvent and a counterion in the gas phase is expected to drive intramolecular stabilization, which may not necessarily occur in solution. Hence, care needs to be taken in interpreting the relevance of glycosyl cation structures obtained using MS and characterized by IRIS. Indeed, VT-NMR experiments of mannosyl donor **10** showed the sole formation of β-glycosyl triflate **11** upon activation (Scheme 3C). The participation of the C-4 *O*-acetyl or *O*-methyl groups could not be detected by NMR. However, because the β-glycoside product is obtained upon addition of a nucleophile, the β-glycosyl triflate is not a reactive intermediate via an S_N_2-like pathway. Hence, β-glycoside formation is expected to occur via oxocarbenium ion **12** or an α-triflate intermediate (Scheme 3C).

**Scheme 4:**
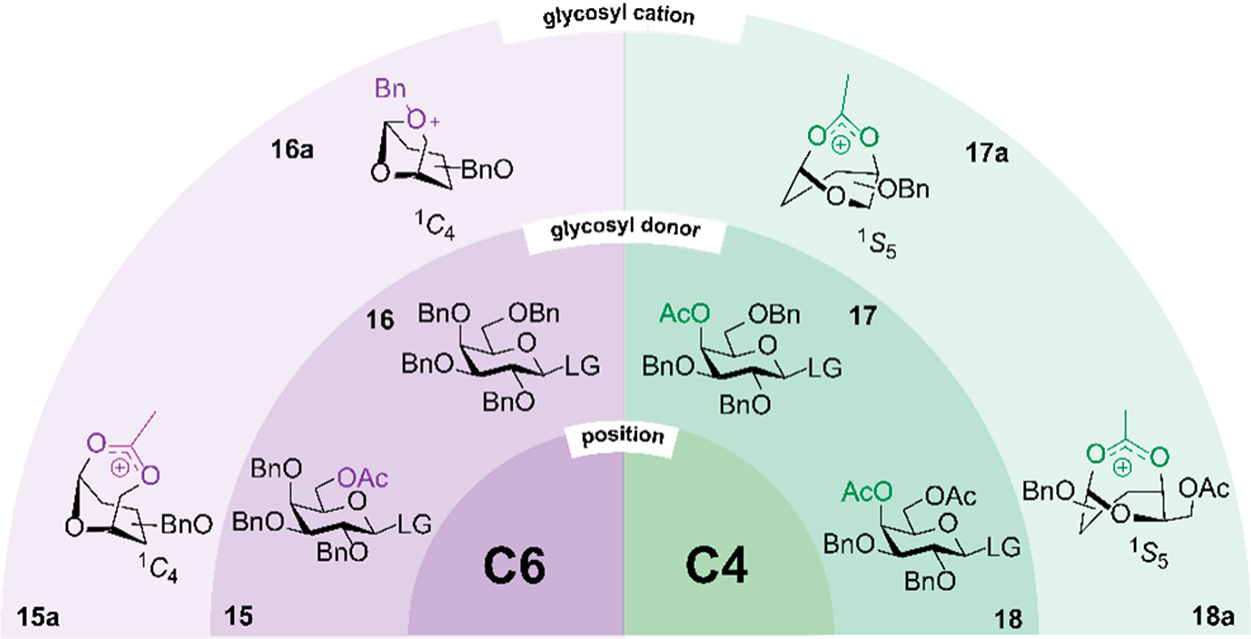
Overview of Characterized Glycosyl Cations Derived from Galactosides Using He Nanodroplet Spectroscopy **15**–**18**, LG = TCAI.

The participation of C-4 acyl groups on the **15**–**18** series of galactoside donors was investigated using the FHI-FEL technique and was reported by Pagel et al.^51^ Galactosides **17** and **18**, each carrying a C-4 acetyl group, formed a bridged glycosyl cation involving the stabilization of the cationic center by C-4 NGP (Scheme 4). Glycosyl cations **17a** and **18a** adopted a ^1^S_5_ ring conformation wherein the C-4-acetyl group participates. In contrast, glycosyl cations formed from C-6 acetylated donor **15** and perbenzylated donor **16** showed unexpected evidence for the presence of one or both oxonium (^1,4^B) and oxocarbenium intermediates with a heavily distorted ring pucker (^5^S_1_). Solution-phase experiments were also performed to investigate the impact of different protecting group combinations on the stereochemical outcome of the glycosylation reaction. Galactosyl imidates carrying a C-4 acetyl group (**17** and **18**) showed a consistently higher α-selectivity than building blocks lacking an acetyl group at this position (**15** and **16**). This observation suggests that the reaction intermediates involved are different. Whether C-4 acyl participation occurs in the condensed phase remains to be investigated.^12^ In a recent low-temperature NMR study reported by Crich et al., only the glycosyl triflate intermediate was found upon activation of a 4-*O*-benzoyl galactopyranosyl donor. Methylation at the 4-position resulted in a more conformationally labile tertiary ester, effectively lowering the barrier to participation, and only in this case was participation of the C-4 ester observed.^54^

**Scheme 5:**
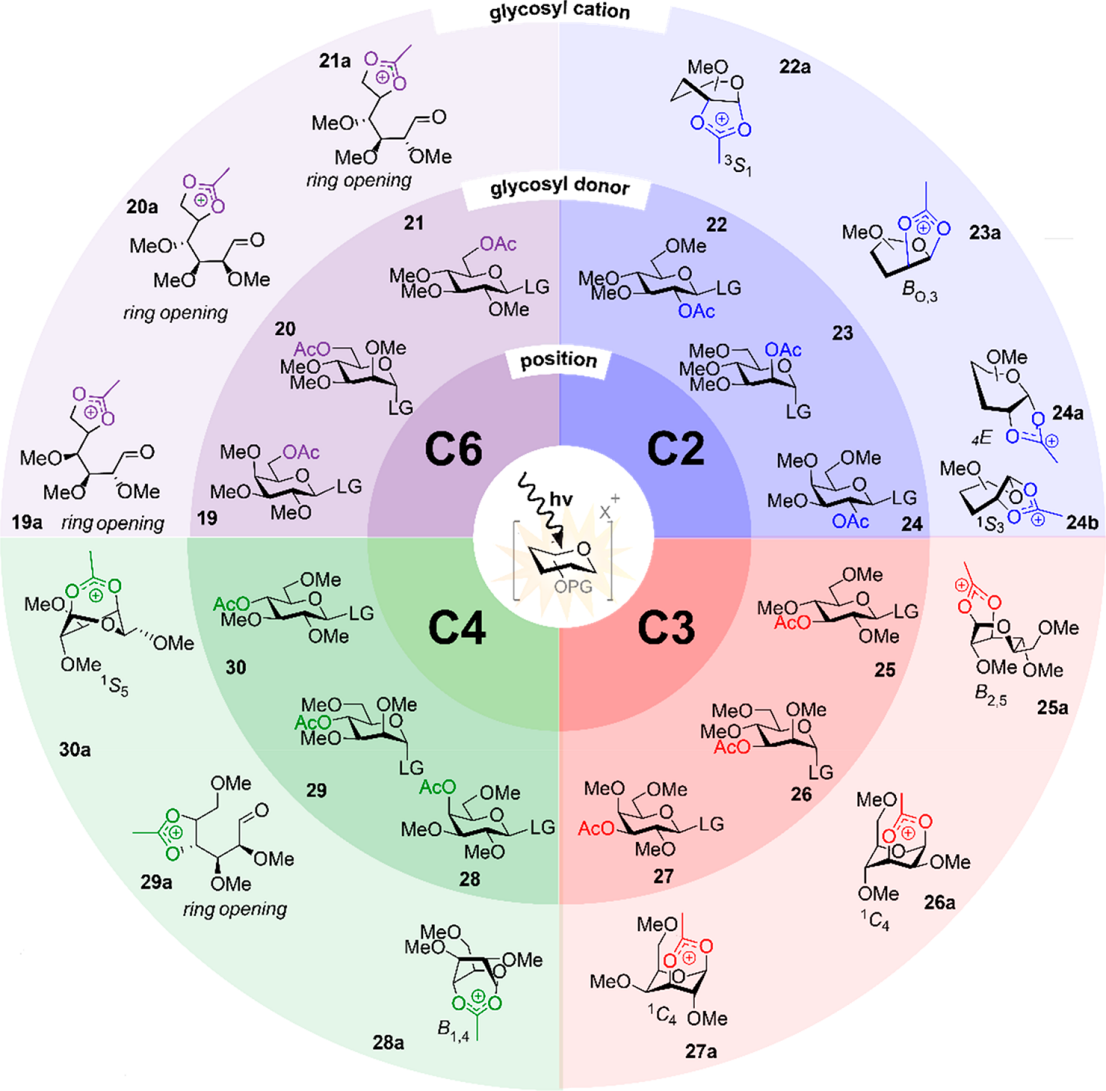
Overview of Glycosyl Cations Characterized from Monoacetyl Glycoside Precursors **22**–**24**, LG = SEt; **25** and **30**, LG = SPh; **26**–**29**, LG = SOPh.

To systematically investigate the role of acyl groups on the glycosyl donor in the stabilization of glycosyl cations, gluco-, galacto-, and mannosyl donors **22**–**24** carrying a single ester at the C-2, C-3, C-4, or C-6 position were investigated. Pagel et al. reported the structures of C-2 acyl-stabilized glycosyl cations (**22a**, **23a**, and **24a**/**b**), and the exact conformation of the pyranose ring was determined (Scheme 5).^21^ The participation of the C-2 ester in glucose induces a ^3^S_1_ ring conformation (**22a**), and the mannosyl cation adopts a B_0,3_ conformation (**23a**). The structural assignment of the galactosyl cation was impeded by a more congested fingerprint region, but coexisting dioxolanium ions bearing distinct ring conformations ^4^E (**24a**) and ^1^S_3_ (**24b**) were proposed. Subsequently, we characterized the full set of glucose, mannose, and galactose cations substituted with a single acetyl ester at the C-3, C-4, or C-6 position.^3^ IRIS afforded highly diagnostic spectra because acetyl participation led to the disappearance of its C=O stretch in the IR spectrum. The participation of the C-3 position was observed for all donors, leading to the formation of glycosyl dioxanium ions (**25a**–**27a**, Scheme 5). In contrast, the IRIS spectra of glycosyl cations derived from donors carrying a C-6 ester all featured a C=O stretch. However, a dioxolanium ion signature was also observed, which is inconsistent with the formation of an unstabilized oxocarbenium ion. Careful analysis using DFT calculations indicated that ring opening had likely occurred by the participation of the C-6 ester at C-5. This affords a dioxolanium ion signature and a C=O stretch corresponding to the C-1 aldehyde (Scheme 5). Participation of the C-4 acetyl ester was observed for glucoside **30** and galactoside **28**, but ring opening was observed for mannoside **29** (Scheme 5).

**Scheme 6:**
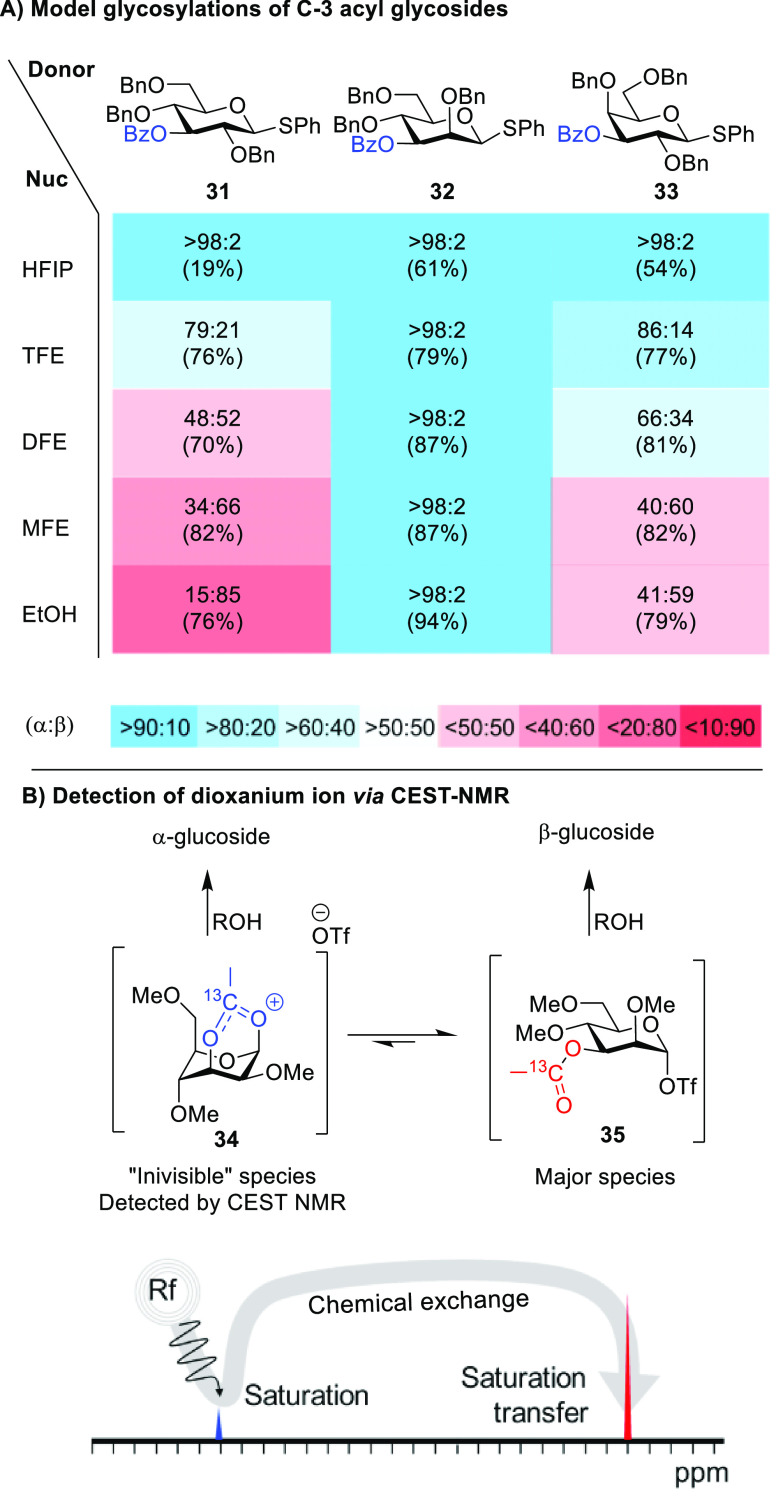
(A) Experimentally Determined Stereoselectivities for Model Glycosylations of C-3 Acyl Glycosides and (B) Detection of the Dioxanium Ion via CEST-NMR HFIP = 1,1,1,3,3,3-hexafluoro-2-propanol, TFE = 2,2,2-trifluoroethanol, DFE = 2,2-difluoroethanol, and MFE = 2-fluoroethanol.

The relevance of the observed gas-phase glycosyl cations may be debated because gas-phase conditions drive the internal stabilization via LRP, which may not necessarily occur under typical glycosylation conditions. Moreover, ions characterized by IRIS likely represent the most stable ions but give little information about the access to other low-energy structures that are more accessible in the condensed phase. To bridge the gap between the gas phase and solution, a full conformational energy landscape (CEL) of all glycosyl cations in their unstabilized (oxocarbenium ion) and stabilized (ester participation) forms was calculated for both gas-phase and solution-phase conditions.^3,55^ The CEL maps revealed that for the mannosyl cation carrying a C-3 *O*-acetyl ester, the energy difference between the oxocarbenium and dioxanium ion forms was greatest, whereas they were close in conformational space. Hence, because of these factors, the strength of internal stabilization was expected to be high for mannose but smaller for the glucose and galactose derivatives. Consistent with this hypothesis, glycosylations with C-3 *O*-benzoyl mannosides were found to be highly α-selective irrespective of the nucleophile strength (Scheme 6A).^56^ Even though C-3 participation was observed in the gas phase for glucoside **25** and galactoside **27**, the extent of stabilization by DFT calculations was moderate and is also reflected in more aselective glycosylation reactions (Scheme 6A).

Hence, only for mannose do we expected dioxanium ion **26a** to play a role in the glycosylation reaction. Recently, we were able to demonstrate the presence of the mannosyl dioxanium ion (**34**) in solution via chemical exchange saturation transfer NMR and establish its exchange kinetics with respect to the α-glycosyl triflate (Scheme 6B).^55^ The kinetics are consistent with a reaction operating under the Curtin–Hammett principle because the interconversion between the dioxanium ion and α-glycosyl triflate occurs much faster than the reaction of α-triflate with the nucleophile. This not only confirms the relevance of the glycosyl cation in glycosylation reactions but also shows how selectivity can be achieved through the formation of the dioxanium ion, as was suggested earlier on the basis of IRIS and CEL maps.

The case of the C-3 *O*-acyl mannosides illustrates that the powerful combination of ion spectroscopy, DFT calculations, and solution-phase experiments goes beyond probing the structure of reactive intermediates. Through careful interpretation, this combination can also provide boundary conditions as to what intermediates can be expected in solution. It is therefore anticipated that the application of ion spectroscopy will be extended to other relevant reactive species.^34,57−59^ Also, the emergence of more sophisticated spectroscopy schemes and hyphenation separates these isomers on the basis of their collisional cross-sections.^60,61^ Alternatively, more sophisticated spectroscopic schemes can be employed to quantify and disentangle coexisting isomers by isomer-selective laser dissociation using vibrational bands that are isomer-specific.^62^ The combined efforts of these different approaches will provide a comprehensive understanding of reaction mechanisms and can provide guidelines for the development of new synthetic strategies.
